# MicroRNA-1269b inhibits gastric cancer development through regulating methyltransferase-like 3 (METTL3)

**DOI:** 10.1080/21655979.2021.1909951

**Published:** 2021-04-05

**Authors:** Jian Kang, Xu Huang, Weiguo Dong, Xueying Zhu, Ming Li, Ning Cui

**Affiliations:** a Department of Gastroenterology, Renmin Hospital of Wuhan University, Wuhan Hubei Province, China; b Department of General Practice, Renmin Hospital of Wuhan University, Wuhan Hubei Province, China

**Keywords:** Gastric cancer, miR-1269b, METTL3, proliferation, metastasis

## Abstract

The dysregulation of microRNAs (miRNAs) expression is relevant to the progression of many tumors. As reported, the abnormal expression of miR-1269b is pivotal in certain cancers’ progression. This work was designed to study the role and hidden mechanism of miR-1269b in gastric cancer (GC) progression. In this work, we proved that miR-1269b was lowly expressed in GC tissues and cell lines, which was associated with larger tumor size and lymph node metastasis. MiR-1269b overexpression repressed the multiplication, migration and invasion of GC cells while miR-1269b inhibition had the opposite effects. Methyltransferase-like 3 (METTL3) was identified as the direct target of miR-1269b in GC cells, and its overexpression reversed the inhibitory effect of transfection of miR-1269b mimics on GC cell viability, migration and invasion. On all accounts, these data indicated that miR-1269b inhibits GC progression via targeting METTL3.

## Graphical abstract

**Figure:**
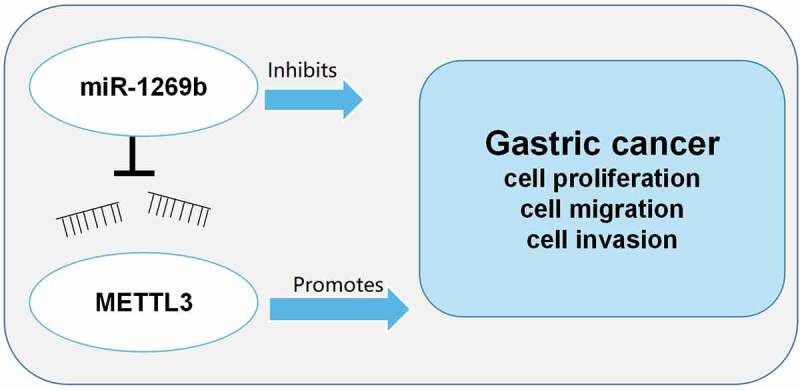

## Introduction

Gastric cancer (GC) is one of the most prevailing malignancies of the digestive system and a leading cause of cancer-related deaths in the globe [1]. Around 50% of GC patients in the world are distributed in East Asia, and the incidence of GC in China is about 6 times that of the United States, leading to huge economic and public health burden [2]. In recent years, as new strategies for diagnosis and treatment of GC evolves, the morbidity and mortality of GC are steadily declining [3]. However, due to tumor metastasis and recurrence, the overall 5-year survival rate of GC patients is still lower than 29% [4]. It is vital to delve into the potential molecular mechanism of GC progression and develop more effective treatments.

MicroRNA (miRNA), recognized as a single-stranded small RNA with a length of about 22*nt*, is highly conserved in evolution and can partake in diverse vital biological processes such as cell growth, differentiation, apoptosis and angiogenesis [5,6]. The abnormal expression of miRNA contributes to cancer progression [7]. Many studies have shown that abnormally expressed miRNAs feature prominently in GC progression [8,9]. For instance, miR-532 is highly expressed in GC, and it can promote migration and invasion of GC cells by targeting NKD1 [8]; miR-4268 inhibits GC cell multiplication by negatively regulating Rab6B expression and inhibiting AKT/JNK signaling pathway [9]; miR-1269b is reported to be highly expressed in various human tumors (such as hepatocellular carcinoma and non-small cell lung cancer), which is relevant to the multiplication, metastasis and drug resistance of tumor cells [10,11]. Instead, the role of miR-1269b in GC is still inconclusive.

N^6^-methyl adenosine (m6A) is the most abundant and prevalent epigenetic modification of mRNA in mammals. It is reported that there are nearly 7600 mRNAs and more than 300 non-coding RNAs modified by m6A in human [12]. m6A is a dynamically reversible RNA methylation modification that is co-regulated by the methyltransferase [methyltransferase-like 3 (METTL3), WTAP, METTL14] complex, demethylases (FTO, ALKBH5) and the corresponding reading proteins (YTHDF, YTHDC) [13]. METTL3 has been proved to be markedly highly expressed in lung cancer, liver cancer and GC, and is involved in regulating the progression of tumors [14–16]. However, how METTL3 works in GC remains inconclusive.

In the present study, miR-1269b expression was found to be down-regulated in GC through microarray analysis. In addition, miR-1269b was unveiled to have a binding site with METTL3 3ʹUTR. Therefore, we hypothesized that miR-1269b could play a key role in GC progression by targeting and regulating METTL3. This study was executed to investigate the expression and clinical significance of miR-1269b in GC and to further explore the mechanism of miR-1269b and MELLT3 in regulating the malignancy of GC cells.

## Methods and materials

### Clinical samples

Forty-three pairs of GC tissues and adjacent tissues from GC patients receiving radical gastrectomy in Renmin Hospital of Wuhan University were stored in liquid nitrogen immediately after surgical resection for further analysis. All of the GC specimens were histopathologically confirmed. All patients had not undergone preoperative radiotherapy or chemotherapy, and had not taken targeted therapies before the surgery. This study, with signed informed consent, was accomplished under the approval and guidance of the Ethics Review Board of Renmin Hospital of Wuhan University.

### Cell culture and transfection

293 T cell, human normal gastric mucosa epithelial cell line GES-1 and human GC cell lines (NCI-N87, SNU-16, AGS and HGC27) were available from the American type culture collection (ATCC; Manassas, VA, USA). Notably, the cells were cultured in DMEM with 10% fetal bovine serum (FBS; Gibco, Grand Island, NY, USA) and 100 U/mL penicillin and 0.1 mg/mL streptomycin (Sigma, St.Louis, MO, USA) with 5% CO_2_ at 37°C.

MiR-1269b mimic (miR-1269b), mimics control (miR-NC), miR-1269b inhibitor (miR-1269b-in), inhibitors control (miR-in), empty plasmid (Vector), pcDNA3.1-METTL3 (METTL3), small interfering RNA (siRNA) targeting METTL3 (si-METTL3) and its negative control (si-NC) were obtained from GenePharma (Shanghai, China). NCI-N87 and SNU-16 cells were transfected with Lipofectamine 3000 reagent (Invitrogen, Carlsbad, CA, USA). 48 h later, cells were immediately collected for the subsequent study.

### Quantitative real-time PCR (qRT-PCR)

Total RNA was extracted from tissues and cell lines by TRIzol reagent (Invitrogen, Carlsbad, CA, USA) according to the instructions. RNA was reversely transcribed into cDNA by PrimeScript RT kit (TaKaRa, Dalian, China). Generally, SYBR Premix Ex Taq (Takara, Dalian, China) was adopted to perform qRT-PCR on an ABI 7500 Fast Real-Time PCR System (Applied Biosystems, Foster City, CA, USA), with U6 and β-actin as endogenous controls of miR-1269b and METTL3, and 2^−ΔΔCT^ method was used for obtain the relative expression levels. The primer sequences are detailed in Table 1.

**Table 1.** Primers for qRT-PCR

**Genes**	**Primers**
miR-1269b	RT primer: 5ʹ-GTCGTATCCAGTGCAGGGTCCGAGGTGCACTGGATACGACCCAGTAGC-3′ F: 5ʹ-TGCGCTGGACTGAGCCATGC-3’
U6	F: 5ʹ-TGCGGGTGCTCGCTTCGGCAGC-3＇ R: 5ʹ-CCAGTGCAGGGTCCGAGGT-3’
METTL3	F: 5′-AAGCTGCACTTCAGACGAAT −3′
	R: 5′-GGAATCACCTCCGACACTC −3′
β-actin	F: 5ʹ-TGAGAGGGAAATCGTGCGTGAC-3’
	R: 5ʹ-AAGAAGGAAGGCTGGAAAAGAG-3’
Abbreviation: F stands for forward; R stands for reverse; RT stands for reverse transcription.	

### Cell counting kit-8 (CCK-8) assay

After transfection, the cells (5 × 10^3^ cells/well) were subsequently inoculated into 96-well plate, and 10 μl of CCK-8 (Beyotime Biotechnology, Shanghai, China) reagent was loaded into the well at the 12^nd^, 24^th^, 48^th^, 72^nd^ and 96^th^ h, and further incubated for 2 h. Ultimately, the optical density at 450 nm was recorded by a microplate reader.

### Wound healing assay

NCI-N87 and SNU-16 cells were inoculated in a 6-well plate. After the cell confluency reached about 90%, a scratch was created with a 200 μL pipette tip and the floating cells were washed with PBS. Then, DMEM without FBS was loaded to the plate. An inverted optical microscope (×200) (Nikon Eclipse Ti Microscope, Tokyo, Japan) was adopted to monitor the wound at 0 and 24 h.

### Transwell migration and invasion assay

Cell migration and invasion were examined by transwell chambers (8 μM pore size, Costar, Cambridge, MA, USA). Generally, transwell chambers pre-coated with Matrigel (Corning, Corning, NY, USA) were used for the invasion assay, but in migration assay, Matrigel was not used. About 5 × 10^4^ cells were suspended in 200 μl of serum-free medium and dripped into the upper chamber. In the lower chamber, 600 μl of medium with 10% FBS was added, and then the cells were cultured at 37°C and 5%CO_2_ for 24 h. Transwell chambers were immediately removed, and the cells remaining in the upper chamber were gently wiped off with a cotton swab. The migrated and invaded cells fixed with methanol for 10 min were stained with 0.1% crystal violet solution for 40 min. 5 fields (×200) were randomly selected under the microscope, and cells were photographed and counted.

### Dual-luciferase reporter gene assay

The wild type (WT) fragment of METTL3 3ʹ-UTR containing miR-1269b binding sequence was cloned into pmirGLO vector (Promega, Madison, WI, USA) to construct METTL3-WT. Then, the target-binding sequence between METTL3 and miR-1269b was mutated, and the mutant type (MUT) fragment was cloned into pmirGLO vector to construct METTL3-MUT. METTL3-WT or METTL3-MUT and miR-1269b mimics or miR-NC were accordingly co-transfected into NCI-N87 cells with Lipofectamine 3000. 48 h later, the luciferase activity was probed by the dual-luciferase reporter gene detection system (Promega, Madison, WI, USA) as protocols.

### Western blot analysis

Cells were lysed by RIPA buffer containing protease inhibitor (Sigma, St. Louis, MO, USA), by which the total proteins were harvested. Protein extracts were quantified by bicinchoninic acid kit (Beyotime, Shanghai, China) and separated by SDS-PAGE, and transferred to polyvinylidene fluoride membrane (Millipore, Billerica, MA, USA). The membranes were then blocked with 5% defatted milk at room temperature for 1 h. After that, the membrane was firstly incubated with specific primary antibody at 4°C overnight and rinsed with TBST, and secondly incubated with corresponding horseradish peroxidase (HRP) coupled anti-rabbit secondary antibody (Sigma, St. Louis, MO, USA) at 37°C for 1 h. Finally, the bands were subsequently visualized by enhanced chemiluminescence (ECL) Plus kit (Beyotime, Shanghai, China) and analyzed by Image Lab software, with β-actin as internal reference. Specifically, the primary antibodies were purchased from Abcam (Shanghai, China): anti-METL3 antibody (ab195352, 1:1000) and anti-β-actin antibody (ab8227, 1:1000).

### Statistical analysis

SPSS 17.0 software (SPSS, Chicago, IL, USA) was applied for statistical analysis, with measurement data presented in the form of ‘mean ± standard deviation’. The difference between two groups was examined by Student’s *t* test, and the difference among multiple groups was examined by one-way analysis of variance with Tukey or Dunnett post hoc test. Besides, the correlation between miR-1269b expression and METTL3 mRNA expression was assessed by Pearson correlation analysis. The counting data were expressed in a contingency table, and χ2 was adopted to analyze the difference between the two groups. All assays were conducted at least in triplicate. Statistically, *P* < 0.05 is meaningful.

## Results

This study was designed to delve into the expression characteristics of miR-1269b in GC tissues and cells, and to analyze the correlation between miR-1269b expression and GC clinicopathological indicators. Additionally, we studied how miR-1269b regulated GC cell proliferation, migration and invasion *in vitro* and its underlying mechanism. This work verified that miR-1269b level was reduced in GC tissues. Transfection of miR-1269b mimics suppressed the growth, migration and invasion of GC cells, while the inhibition of miR-1269b had the opposite effects. Furthermore, it was confirmed that miR-1269b participated in restraining the multiplication, migration and invasion of GC cells by targeting and inhibiting METTL3.

### The expression and clinical significance of miR-1269b in GC

**Figure 1.**
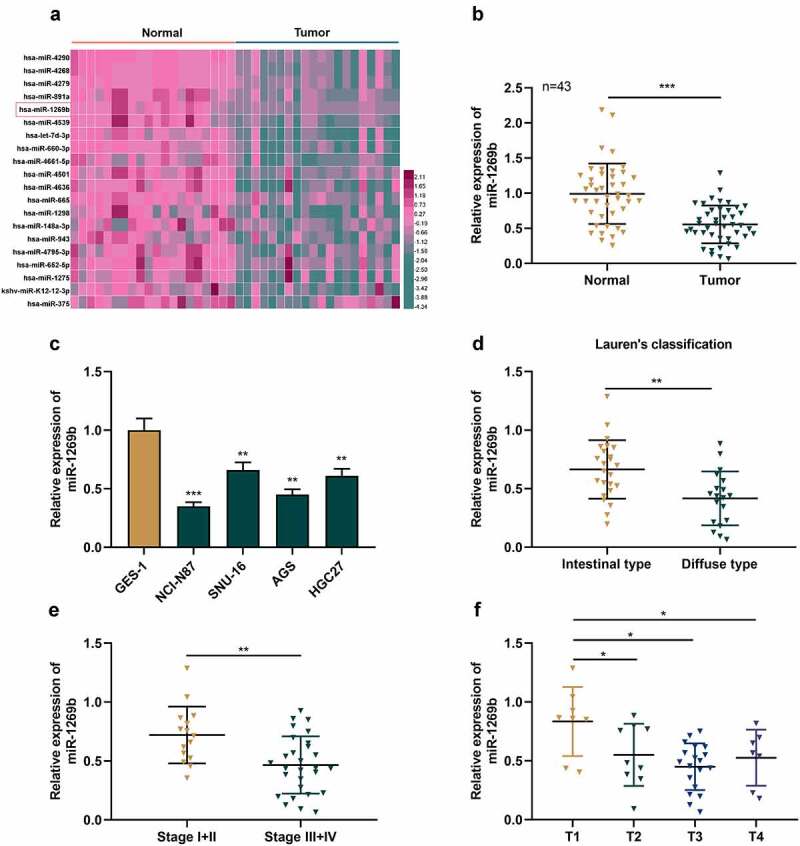
***The expression of miR-1269b is inhibited in GC tissues and cells***(a) Expression levels of miRNAs in GC tissues (Tumor) and adjacent normal tissues (Normal) in GSE93415. (b) The expression of miR-1269b in GC tissues and adjacent tissues was detected by qRT-PCR. (c) The expression of miR-1269b in GC cells and GES-1 cells was detected by qRT-PCR. (d) Difference in the expression level of miR-1269b between intestinal type GC tissues and diffuse type GC tissues. (e and f) The expression of miR-1269b in GC tissues with different clinical stage (e) and T stage (f). *P < 0.05, **P < 0.01, and ***P < 0.001

By analyzing the microarray dataset GSE93415 (https://www.ncbi.nlm.nih.gov/) in Gene Expression Omnibus database, we observed that miR-1269b expression was inhibited in GC tissues as against normal tissues (Figure 1 A). qRT-PCR uncovered that miR-1269b expression in GC tissues was markedly lower than that in adjacent tissues (Figure 1 B). Relative to normal gastric epithelial cell line GES-1, miR-1269b expression in four GC cells was down-regulated (Figure 1 C), suggesting that miR-1269b may be related to the pathogenesis of GC. Moreover, compared with intestinal type, miR-1269b expression was markedly lower in diffuse type cancer tissues of GC patients (Figure 1 D). MiR-1269b expression was lower in GC tissues of higher clinical stages (Stage III and stage IV) as against GC tissues of early clinical stages (Stage I and stage II) (Figure 1 E). MiR-1269b expression was also lower in T1 GC tissues compared with T2&3&4 GC tissues (Figure 1 F). Consistently, chi-square test also proved that low miR-1269b expression was associated with larger tumor size and lymph node metastasis of GC patients (Table 2).

**Table 2.** The association of miR-1269b expression with clinicopathological parameters

**Characteristics**	**Number of patients** **(n = 43)**	**miR-1269b expression**		***χ^2^***	***P* value**
		**Low (n = 23)**	**High (n = 20)**		
Gender					
Male	24	13	11	0.0100	0.9202
Female	19	10	9		
Age(years)					
<61	23	10	13	1.9917	0.1582
≥61	20	13	7		
Lymph node invasion					
Negative	17	5	12	6.5511	0.0105*
Positive	26	18	8		
Drinking alcohol					
Yes	23	15	8	2.7344	0.09820
No	20	8	12		
Tumor size (cm)					
<5	14	4	10	5.1804	0.0228*
≥5	29	19	10		
Differentiation					
Well-moderately	15	9	6	0.3926	0.5309
Poorly	28	14	14		
Distant metastasis					
Yes	26	14	12	0.0034	0.9536
No	17	9	8		

Note: * *P* < 0.05.

### MiR-1269b inhibits the malignant biological behaviors of GC cells

**Figure 2.**
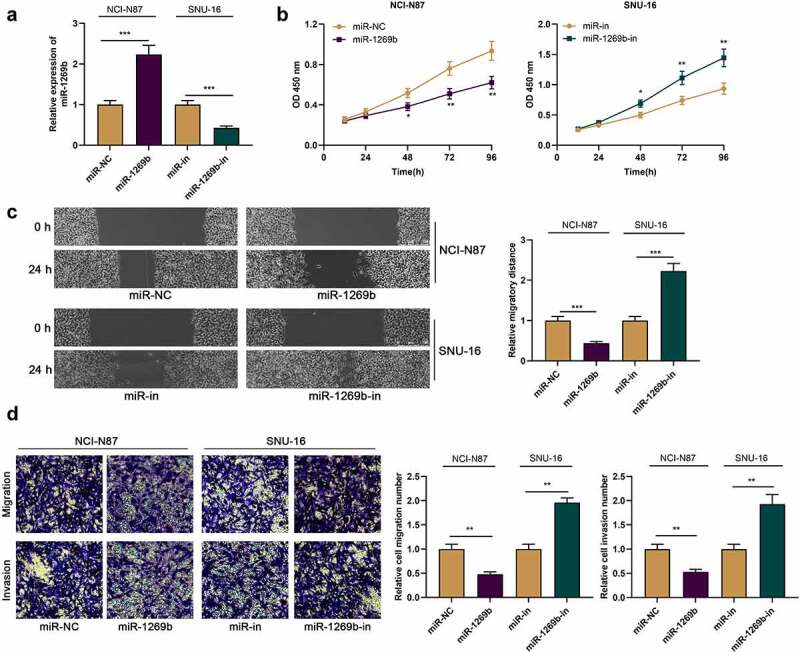
***miR-1269b inhibits the proliferation, migration and invasion of GC cells*** (a) miR-1269b expression in NCI-N87 or SNU-16 cells transfected with miR-1269b mimics or inhibitors were detected by qRT-PCR. (b) CCK-8 assay was used to evaluate cell proliferation. (c) The migration ability of cells was evaluated by wound healing assay. (d) The migration and invasion ability of cells were evaluated by transwell assay. *P < 0.05, **P < 0.01, and ***P < 0.001

We then transfect miR-1269b mimics in NCI-N87 cells with the lowest expression of miR-1269b, and transfect miR-1269b inhibitors in SNU-16 cells with the highest expression of miR-1269b, with qRT-PCR verifying the transfection efficiency (Figure 2 A). CCK-8 assay uncovered that relative to the control, miR-1269b overexpression repressed the multiplication of NCI-N87 cells, while miR-1269b down-regulation promoted the viability of SNU-16 cells (Figure 2 B). Scratch healing assay and transwell experiment proved that miR-1269b overexpression significantly blocked cell migration and invasion, while its down-regulation worked oppositely (Figure 2 C and D).

### METTL3 is the downstream target of miR-1269b in GC cells

**Figure 3.**
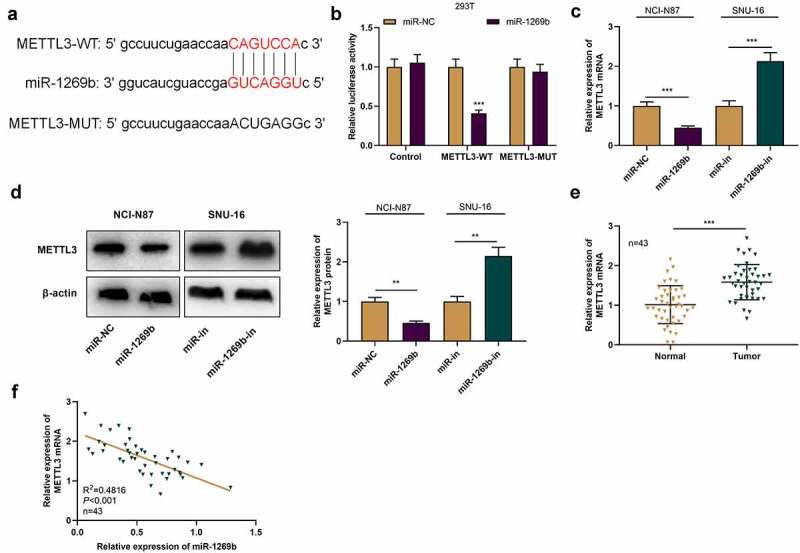
***METTL3 is the target of miR-1269b*** A) The predicted binding sequence between METTL3 mRNA 3ʹUTR and miR-1269b. (b) Dual-luciferase reporter gene assay was used to verify the binding relationship between METLL3 mRNA 3ʹ UTR and miR-1269b. (c) and (d) qRT-PCR and Western blot were used to detect the expression of METTL3 mRNA and protein in NCI-N87 and SNU-16 cells transfected with miR-1269b mimics or inhibitors. (e) qRT-PCR was used to detect the expression of METTL3 mRNA in GC tissues and adjacent tissues. (f) Pearson correlation was used to analyze the correlation between METTL3 mRNA expression and miR-1269b expression in GC tissues. **P < 0.01, and ***P < 0.001

To decipher the hidden mechanism of miR-1269b in GC progression, we predicted the downstream target of miR-1269b through StarBase online analysis database (http://starbase.sysu.edu.cn/), and found that there was a binding site between miR-1269b and METTL3 mRNA 3ʹ-UTR (Figure 3 A). Dual-luciferase reporter gene assay highlighted that miR-1269b overexpression could demonstrably reduce the luciferase activity of METTL3-WT in 293 T cells, but that of METTL3-MUT was not affected significantly (Figure 3 B). qRT-PCR and Western blot suggested that as against the control (miR-NC or miR-in), miR-1269b overexpression significantly reduced METTL3 mRNA and protein expressions in NCI-N87 cells while transfection with miR-1269b inhibitors functioned oppositely in SNU-16 cells (Figure 3 C and D). With qRT-PCR, we also discovered that METTL3 expression was markedly higher in GC tissues than that in adjacent tissues (Figure 3 E). The correlation analysis highlighted that miR-1269b expression was negatively correlated with METTL3 mRNA expression in GC tissues (Figure 3 F). Above results implied that miR-1269b could target METTL3 mRNA 3ʹ-UTR and negatively regulate METTL3 expression in GC cells.

### MiR-1269b restrains the multiplication, migration and invasion of GC cells via targeting METTL3

**Figure 4.**
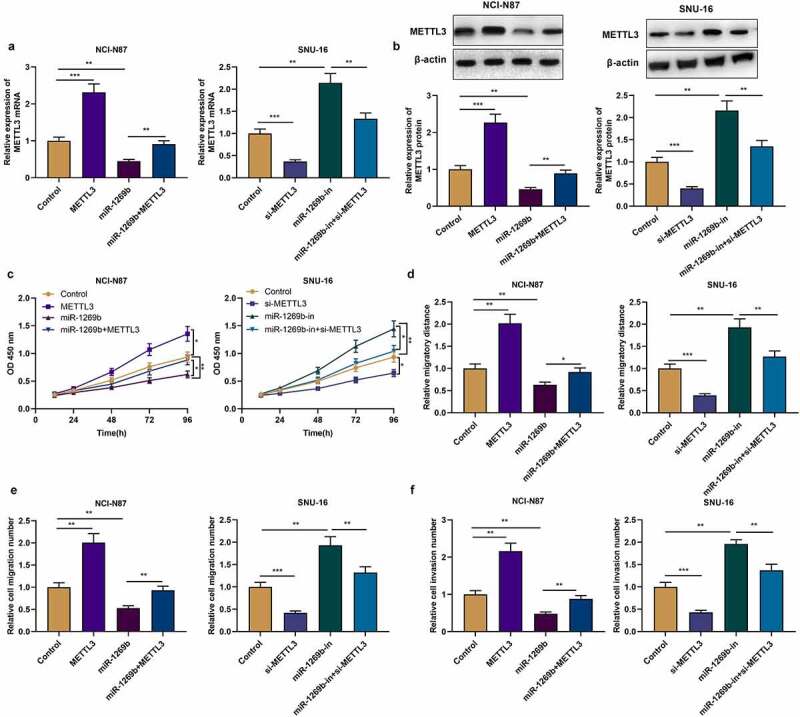
***miR-1269b regulates the progression of GC by targeting METTL3*** (a) and (b) qRT-PCR and Western blot were used to detect the expression of METTL3 mRNA and protein after the transfection. (c) CCK-8 assay was used to evaluate GC cell proliferation. (d)–(f) The migration and invasion ability of NCI-N87 and SNU-16 cells were evaluated by wound healing assay and transwell assay. *P < 0.05, **P < 0.01, and ***P < 0.001

To probe the role of miR-1269b/METTL3 axis in GC, we transfected pcDNA3.1-METTL3, miR-1269b mimics and miR-1269b mimic+pcDNA3.1-METTL3 into NCI-N87 cells, respectively, while si-METTL3, miR-1269b inhibitor and si-METTL3+ miR-1269b inhibitors were transfected into SNU-16 cells, respectively. qRT-PCR and Western blot uncovered that pcDNA3.1-METTL3 transfection could up-regulate the expression of METTL3 in NCI-N87 cells, and weaken the inhibitory effect of overexpression of miR-1269b on METTL3 expression; si-METTL3 transfection reduced METTL3 expression in SNU-16 cells, and the up-regulation of METTL3 expression caused by miR-1269b inhibition was partly reversed (Figure 4 A, B). CCK-8, scratch healing assay and transwell assay were performed to detect the role of miR-1269b/METTL3 axis in GC cell multiplication, migration and invasion. As against the control, we found that METTL3 overexpression facilitated the malignant biological behaviors of NCI-N87 cells, while knocking down METTL3 functioned oppositely in SNU-16 cells; METTL3 overexpression attenuated the effect of overexpression of miR-1269b on the malignant biological behaviors of NCI-N87 cells, while transfection of si-METTL3 weakened effects of miR-1269b-in in SNU-16 cells (Figure 4 C-F). Aforementioned results highlighted that miR-1269b/METTL3 axis is implicated in modulating the multiplication, migration and invasion of GC cells.

## Discussion

In the past decades, there has been a lack of highly sensitive and specific indicators for the diagnosis and prognosis prediction of GC [17]. In recent years, the abnormal expression of various miRNAs in plasma or cancer tissues of GC patients has been reported, and miRNAs are expected to become new molecular markers for the diagnosis of GC [18,19]. Here we discovered that miR-1269b expression was reduced in GC tissues and cell lines, and that miR-1269b underexpression was associated with unfavorable pathological characteristics of GC patients, indicating that miR-1269b has the potential to be the diagnosis and prognosis biomarker of GC.

MiRNA, as an important member of non-coding RNA family, can inhibit gene expression by inducing mRNA degradation or blocking translation [20]. MiRNA, as reported, is implicated in many biological processes, such as cell cycle progression, multiplication, apoptosis and metastasis of cancer cells [21]. MiRNA expression profile is related to the characteristics of diverse cancers and can be used for screening differentially expressed miRNAs [22,23]. Through microarray analysis, we found that the expression of miR-4636, miR-1298, miR-4268, miR-4290, miR-1275, miR-148a-3p, miR-375, miR-943 and miR-665 were down-regulated in GC tissues, which is consistent with the previous research findings [9,24–31]. Herein, we mainly focused on miR-1269b. According to the previous reports, miR-1269b expression is raised in cisplatin-resistant non-small cell lung cancer tissues and cell lines, and can enhance cisplatin resistance of cancer cells via modulating PTEN/PI3K/AKT pathway [11]; miR-1269b can down-regulate SVEP1 expression through PI3K/Akt signaling pathway, thus expediting the multiplication and metastasis of hepatocellular carcinoma cells [32]. Here, we observed that miR-1269b expression in GC tissues and cells was decreased significantly. Functionally, miR-1269b overexpression inhibited the malignant biological behaviors of GC cells, and inhibiting miR-1269b had the opposite effect. Based on our research results, we concluded that miR-1269b may be a tumor suppressor in GC.

METTL3 is first identified as a 70 kDa protein from HeLa cell lysate [33]. Subsequent studies have found that METTL3 contains two domains that bind S-adenosylmethionine (SAM) and catalyze m6A formation and is of the activity of independently catalyzing m6A modification of RNA [34]. In m6A methyltransferase complex, METTL3 is a functional subunit and can form heterodimer with METTL14 [35]. Reportedly, METTL3 is a vital regulator in malignant tumors [36]. For example, METTL3 expression is elevated in breast cancer tissues and cells and can promote the multiplication of breast cancer cells and inhibit apoptosis by targeting Bcl-2 [37]; METTL3 upregulation promotes the metastasis of colorectal cancer cells through miR-1246/SPRED2/MAPK signaling pathway [38]. Importantly, the high expression of METTL3 predicts the poor prognosis of GC patients [16]; another study reports that METTL3 overexpression promotes GC cell multiplication and liver metastasis [39]. MiRNA, as reported, is a paramount regulator of gene expression. The aberrant expression of miRNAs can induce expression change of METTL3 [40–42]. For example, miR-33a blocks the multiplication of NSCLC cells via targeting METTL3 [40]; miR-4429 restrains the progression of GC by targeting METTL3 to inhibit m6A-caused stabilization of SEC62 [42]. In this work, METTL3 was confirmed to be the downstream target of miR-1269b in GC cells, and miR-1269b could negatively regulate METTL3 expression at protein and mRNA levels. We also proved that METTL3 was highly expressed in GC tissues, which is consistent with previous studies [16,39]. Additionally, METTL3 promoted the malignant biological behaviors of GC cells, and attenuated the inhibitory effect caused by miR-1269b overexpression. These findings implied that miR-1269b can restrain proliferation, migration and invasion of GC cells by down-regulating METTL3. However, as mentioned above [11,32], METTL3 is not the only downstream target gene of miR-1269b. In the following studies, it is necessary to investigate whether miR-1269b can regulate the development of GC via other mechanism. In addition, the upstream mechanisms that cause changes in miR-1269b expression are also worthy of being explored.

### Conclusion

To recapitulate briefly, miR-1269b is in low expression in GC tissues and cells, and miR-1269b restrains the multiplication, migration and invasion of GC cells via targeting METTL3. Our work elucidate the important role of miR-1269b/METTL3 pathway in GC development, which provides possible biomarkers and targets for GC.

## Data Availability

The data used to support the findings of this study are available from the corresponding author upon request.

## Highlights

1. MiR-1269b is down-regulated in GC tissues and cells.

2. The low expression of miR-1269b is related to larger tumor size and lymph node metastasis of the patients.

3. MiR-1269b inhibits GC cell proliferation, migration and invasion by targeting METTL3.
